# When Memory and Metamemory Align: How Processes at Encoding Influence Delayed Judgment-of-Learning Accuracy

**DOI:** 10.3390/jintelligence10040101

**Published:** 2022-11-11

**Authors:** Gregory Isaac Hughes, Ayanna Kim Thomas

**Affiliations:** 1 The U.S. Army Combat Capabilities Development Command (DEVCOM) Soldier Center, Natick, MA 01760, USA; 2 The Center for Applied Brain and Cognitive Sciences (CABCS), Medford, MA 02155, USA; 3 Department of Psychology, Tufts University, Medford, MA 02155, USA

**Keywords:** metamemory, judgments of learning, retrieval practice, elaboration

## Abstract

Judgments of learning are most accurate when made at a delay from the initial encoding of the assessed material. A wealth of evidence suggests that this is because a delay encourages participants to base their predictions on cues retrieved from long-term memory, which are generally the most diagnostic of later memory performance. We investigated the hypothesis that different types of study techniques affect delayed JOL accuracy by influencing the accessibility of cues stored in long-term memory. In two experiments, we measured the delayed-JOL accuracy of participants who encoded semantically unrelated and weakly related word pairs through one of three study techniques: reading the pairs twice (study practice), generating keywords (elaborative encoding), or taking a cued-recall test with feedback (retrieval practice). We also measured the accessibility, utilization, and diagnostic quality of two long-term memory cues at the time of the delayed JOL: (a) retrieval of the target, and (b) noncriterial cues (retrieval of contextual details pertaining to the encoding of the target). We found that the accessibility of targets was positively associated with delayed-JOL accuracy. Further, we provide evidence that when study techniques enhance the accessibility of targets, they likewise enhance delayed-JOL accuracy.

## 1. Introduction

When preparing for an exam, students must decide how to allocate study time across items. Making these decisions effectively requires that students can accurately predict what items are most likely to be remembered or forgotten (Metcalfe 2002; Dunlosky and Hertzog 1998). Such predictions are called judgments of learning (JOL). The accuracy of JOLs has been shown to depend on several factors, including (a) the time interval between encoding and the prediction (Nelson and Dunlosky 1991), and (b) how the assessed material was initially encoded (Hughes et al. 2018). In the present research, we investigated the interaction between these two factors.

In a typical JOL paradigm, participants study prompt-target word pairs (e.g., mother–child), predict the likelihood of remembering the target (second word) when later presented with the prompt (first word), and then take a final-memory test (for a review see Rhodes 2016). The accuracy of JOLs is examined by measuring the degree of correspondence between the predictions and performance on the final test. This correspondence has been traditionally examined either by (a) comparing mean JOLs to mean final-test performance (absolute accuracy), or (b) examining item-by-item gamma correlations between JOLs and final-test performance (relative accuracy). Relative accuracy, which is the focus of this study, is perfect when the highest JOLs are always associated with later memory success, and the lowest JOLs are always associated with later memory failure. For brevity, we will refer to the relative accuracy of JOLs simply as JOL accuracy. 

Judgments of learning are most accurate when delayed from the initial encoding of material (the delayed-JOL effect; Nelson and Dunlosky 1991). The major theories regarding this effect all posit that delay benefits JOL accuracy by affecting the cues that participants utilize to make their predictions. Without a delay, people tend to utilize cues that are transiently available during or immediately after encoding. In contrast, interjecting a delay encourages people to use cues that are more stably represented in long-term memory (Nelson and Dunlosky 1991; Spellman and Bjork 1992). The cue that has received the most attention in the delayed-JOL literature is whether a participant can retrieve the target at the time of the prediction (Nelson et al. 2004). The basic idea is that participants express high JOLs when they retrieve the target and low JOLs when they do not, resulting in above-chance JOL accuracy because success or failure in retrieving the target is generally diagnostic of future memory performance. 

### 1.1. The Delayed JOL Effect

All contemporary theories suggest that JOLs and other metacognitive predictions are inferential. According to these theories, people cannot directly assess the strength or durability of their memory traces but must infer these properties by accessing and utilizing available evidence, or *cues* (Bröder and Undorf 2019; Koriat 1997). People use multiple cues when making their JOLs, including the degree of familiarity with the stimulus (Metcalfe et al. 1993; Son and Metcalfe 2005), a priori beliefs about memory (Koriat 1997), and explicit retrieval of information pertaining to the target (Nelson and Dunlosky 1991). Given the inferential nature of JOLs, predictive accuracy therefore depends on the utilization of cues that are diagnostic of later memory.

Other cues likely play a role in delayed JOL accuracy, such as the retrieval of other information about the target (i.e., noncriterial cues; Parks 2007). Evidence for this comes from the literature on similar prospective-memory predictions, like feeling-of-knowing judgments (predictions of remembering presently unrecallable targets). Participants express higher feeling-of-knowing predictions when they consciously retrieve noncriterial cues, such as the emotional valence of a target (Thomas et al. 2011), the imagery used to encode the target (Hertzog et al. 2014), or the gender of the speaker for aurally presented targets (Brewer et al. 2010). Retrieval of noncriterial cues is generally diagnostic of later memory performance, and thus utilizing these cues supports predictive accuracy.

### 1.2. Encoding and Delayed JOL Accuracy

Research suggests that delayed-JOL accuracy depends on how information was initially encoded. For example, Hughes et al. (2018) had college-aged adults encode weakly related word pairs by reading the pairs twice (study practice), generating a keyword (the mediator) that thematically linked paired words (elaborative encoding), or by reading the pairs once and taking a cued-recall test with feedback (retrieval practice). Participants made delayed JOLs after a 48-hour delay and then took a final four-alternative forced-choice test. Retrieval practice and elaborative encoding led to higher delayed-JOL accuracy than study practice. As with delayed-JOLs, feeling-of-knowing accuracy has also been shown to be influenced by encoding processes (Carroll and Nelson 1993; Lupker et al. 1991; Hertzog et al. 2010, 2014; Thomas et al. 2011). For example, Lupker et al. (1991) found that encoding paired associates with a sentence-generation task led to higher FOK accuracy than a shallow vowel-counting task. More recently, Hertzog and colleagues found that increasing the number of study trials enhanced FOK accuracy (Hertzog et al. 2010, 2014).

The purpose of the present study was to explore how study techniques influence delayed-JOL accuracy. We examine three possibilities here, which are not mutually exclusive. First, encoding techniques could, like delay, influence the way that people utilize cues. Perhaps some ways of studying material make some cues more likely to be used relative to others. For example, encoding techniques that focus on and/or enhance the retrieval of targets (e.g., retrieval practice) could make the cue of target retrieval especially salient, and more utilized, compared to encoding methods that do not involve explicit retrieval (e.g., study practice) or those that emphasize the generation of mnemonic devices or keywords (e.g., elaborative encoding). This explanation would account for the findings reported by Hughes et al. (2018). If retrieval practice enhanced the utilization of target retrieval as a cue, then this would explain why it likewise enhanced delayed JOL accuracy, since target retrieval is highly predictive of future memory performance. 

Second, study techniques could affect JOL accuracy by influencing the diagnosticity of retrieval cues. Holding utilization constant, differences in the diagnosticity of those cues could drive differences in JOL accuracy. It is conceivable that the noncriterial cues generated during retrieval practice (e.g., memory for past test performance; Finn and Metcalfe 2008), and elaborative encoding (e.g., the mediator keyword; Dunlosky et al. 2005) would be more task-relevant, and therefore more diagnostic, than those that are spontaneously generated during a control task like study practice.

Third, processes at encoding could affect JOL accuracy by affecting the quantity of diagnostic cues participants can retrieve from long-term memory (i.e., cue accessibility). It stands to reason that for participants to rely on diagnostic cues from long-term memory, then the initial encoding technique must have been sufficiently effective to support the storage and retrieval of these cues. From this perspective, effective study techniques would be more likely to lead to higher levels of metacognitive prediction accuracy than lesser techniques. Indeed, research suggests that the quality of the original encoding event affects metacognitive accuracy.

The proposal that encoding quality is positively related to metacognitive prediction accuracy is not controversial. However, the extent of this relationship has not been adequately explored, and there are methodological and theoretical reasons to examine this association with a higher degree of granularity. One possibility is that encoding quality only matters inasmuch as it yields a single diagnostic cue at the time of the JOL. To illustrate this point, consider two participants who studied 10 items in a hypothetical experiment. When making delayed JOLs, one participant retrieves a diagnostic cue on five of the 10 trials, assigning high JOLs to these items and low JOLs to all others. The other participant retrieves a diagnostic cue only on one trial, and similarly assigns high JOLs to only this single item. On a later test, participants successfully remember the target of only the five items and one item, respectively, thus exhibiting equivalent and perfect delayed-JOL accuracy. This illustration exemplifies the proposal that the relative accuracy of JOLs is logically independent of absolute levels of long-term memory performance (Benjamin and Diaz 2008; Mazzoni and Nelson 1995; Nelson 1984; Gonzalez and Nelson 1996; Spellman et al. 2008). To summarize this view, JOL accuracy is maximized when there is some number of trials with and without the retrieval of diagnostic cues, but differences in the number of retrieved diagnostic cues would not matter (beyond floor and ceiling). This would limit the influence of encoding on JOL accuracy.

Another possibility is that the quality of the original encoding tracks linearly with JOL accuracy in a finer-grained manner. We make the case for this proposal by pointing out that the above illustration makes assumptions that will not always occur. First, the illustration assumes that people always utilize diagnostic over non-diagnostic cues. However, the literature is rife with examples in which non-diagnostic cues are valued highly by participants (e.g., font size; Rhodes and Castel 2008). Thus, they may sometimes express higher JOLs on trials in which a non-diagnostic cue is retrieved compared to one in which a diagnostic cue is retrieved, therefore reducing JOL accuracy. The overall quantity of diagnostic cues retrieved from long-term memory could therefore preserve JOL accuracy by reducing the probability that participants will resort to the use of attractive but low-value alternatives. Second, the illustration assumes perfect diagnosticity of the retrieved cues. However, even the cue of target retrieval can be imperfect. Participants sometimes fail to retrieve a target but still remember it on a later cued-recall test (Metcalfe et al. 1993; Jameson et al. 1990; Reder and Ritter 1992) or recognition test (Koriat 1993; Hertzog et al. 2013). When participants fail to retrieve a target, they might express a low JOL, but still remember the target on a later test, reducing JOL accuracy. Consequently, the more often participants retrieve targets when making their JOLs, the less prone they will be to that metacognitive mishap. 

**Figure 1:**
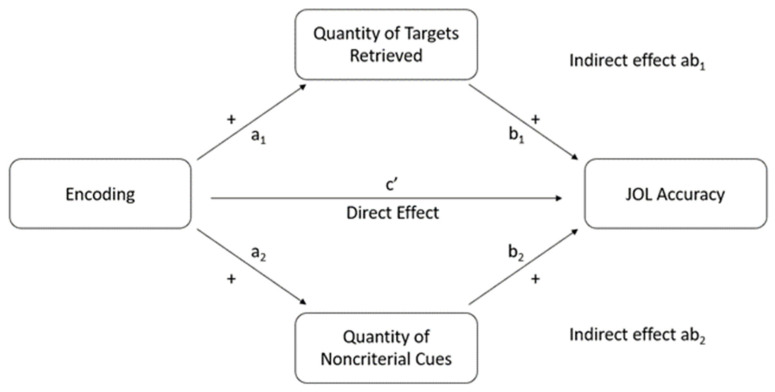
Proposed mediation model of encoding technique on JOL accuracy through two mediators, the quantity of targets retrieved (Mediator 1) and noncriterial cues retrieved (Mediator 2). The model assumes that encoding influences each mediator, and that each mediator is positively associated with JOL accuracy. Letters with subscripts denote a coefficient in the path analysis.

We hypothesized that encoding techniques would influence delayed-JOL accuracy by affecting the quantity of diagnostic cues retrieved from long-term memory. This proposed mediation model is depicted in Figure 1.

### 1.3. The Current Study

**Figure 2:**
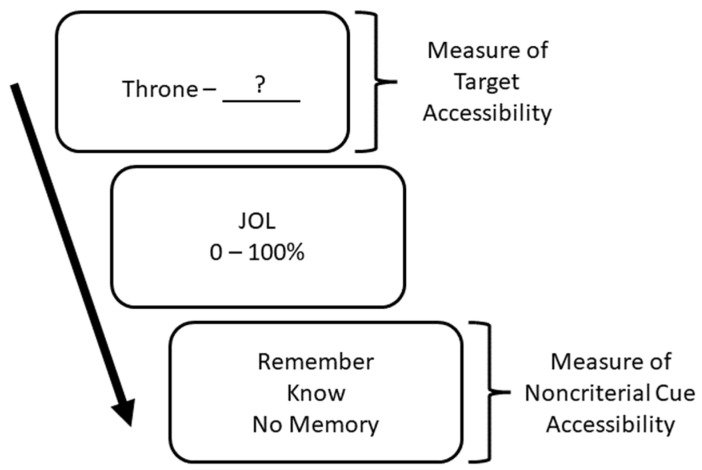
Depiction of the JOL Phase. For each word pair, participants made a cued-recall attempt, a JOL, and a remember/know/no memory response. Participants completed each of these three tasks before proceeding to the next word pair.

In two experiments, we used an adapted version of the paradigm used by Hughes et al. (2018). Participants learned 20 weakly related (e.g., Throne—Castle) and 20 unrelated (e.g., Allergy—Divorce) word pairs either with study practice, elaborative encoding, or retrieval practice with feedback. After a 48-hr retention interval, participants made trial-by-trial JOLs. Each JOL trial consisted of three parts (see Figure 2). First, participants made a cued-recall attempt of the target (e.g., Throne—?). Second, participants made their JOLs, in which they estimated the probability of selecting the target word on an upcoming four-alternative multiple-choice test on a 0 to 100% scale. Third, they indicated whether the prompt word of the paired associate evoked a remember, know, or no memory response. Accessibility of noncriterial cues was operationalized as participants producing a “remember” response when they could not retrieve the target. After making JOLs on all items, participants took a four-alternative forced-choice test. This design allowed us to examine all three possible ways in which different types of encoding can influence delayed-JOL accuracy—by affecting cue accessibility, cue utilization, and/or cue diagnosticity. 

Although the measurement of noncriterial cues is frequently accomplished by asking participants to retrieve a specific type of information (e.g., the valence of the target), we instead adopted the remember/know task (Tulving 1985) to cast a broader net to capture the wide variety of possible noncriterial cues that can be recalled. In a remember/know task, participants indicate whether a given stimulus evokes the retrieval of contextual details pertaining to the initial encoding event (remember), merely a feeling of familiarity without the retrieval of contextual details (know), or nothing at all (no memory). In an episodic feeling-of-knowing experiment, Isingrini et al. (2016) employed the remember/know procedure to measure the retrieval of noncriterial cues. In their experiment, participants encoded word pairs and after a retention interval were asked to retrieve the target when given the prompt word. When a participant could not retrieve the target, they made a feeling-of-knowing prediction and then indicated whether the prompt word evoked a remember, know, or no memory response. The authors conceptualized the retrieval of noncriterial cues as occurring on trials in which participants produced a “remember” response but could not retrieve the target. The advantage of using the remember/know task in this way is that it can capture a wide range of noncriterial cues; there are myriad types of noncriterial cues and measuring all of them individually is difficult because they can be idiosyncratic.

We hypothesized that retrieval practice and elaborative encoding would lead to higher delayed-JOL accuracy than study practice. Further, we hypothesized that differences in JOL accuracy across groups would be mediated by the accessibility of both targets and noncriterial cues. That is, we expected that retrieval practice and elaborative encoding would increase the accessibility of these cues, and that the accessibility of these cues would be positively-associated with JOL accuracy. 

Finally, we hypothesized that the influence of study techniques on JOL accuracy would depend on the associative relatedness of word pairs. Therefore, in addition to the weakly related word pairs used by Hughes et al. (2018), we also had participants study unrelated word pairs. We hypothesized that the effects of study techniques on (a) JOL accuracy and, (b) accessibility of cues, would be greater with weakly related compared to unrelated word pairs. This hypothesis was based on the idea that, within the time limit allotted per item, retrieval practice and elaborative encoding would be easier to complete for weakly related compared to unrelated items. That is, we expected that it would be easier to retrieve a target (Bulevich et al. 2016) or generate a mediator (Paivio et al. 1988) during encoding of weakly related compared to unrelated word pairs. Because the memorial benefits of retrieval practice (Pyc and Rawson 2009) and elaborative encoding (Kane and Anderson 1978) hinge on the successful completion of the task, we expected these techniques to be most effective with weakly related word pairs. Thus, relative to study practice, the advantage of retrieval practice and elaborative encoding on JOL accuracy, encoding, and retention should be greatest with weakly related items.

Because we used a recognition test as the final measure of memory, it was important to include a way to account for the contamination of correct guessing on the memorial and metamemorial measures. This is because correctly guessing the answer on the final test by pure chance can artificially reduce JOL accuracy (Leonesio and Nelson 1990; Dunlosky et al. 2016; Schwartz and Metcalfe 1994). When a participant has no memory at all for an item, they are likely to express a low JOL, and if they subsequently guess the correct answer on the final memory test by pure chance, then this would mean that a low JOL is paired with memory success, reducing metamemorial accuracy. This reduction in JOL accuracy is artificial because this hypothetical participant genuinely knew they had no memory for the item in question and their low JOL was appropriate. Consequently, their low JOL response should not deflate their measure of metacognitive accuracy, but rather should inflate it because their low JOL was appropriate. The contamination of correct guessing is especially problematic when comparing JOL accuracy across groups or conditions that vary in memory performance, because the greater the number of trials that a participant has no memory of an item, the more opportunities there are for a correct guess to occur. We therefore included a confidence judgment on the final test with an option to express “complete guess.” One way to examine the influence of correct guessing is to recode all items as false that participants stated their response was a complete guess (e.g., Thiede and Dunlosky 1994).

## 2. Experiment 1

### 2.1. Materials and Methods

#### 2.1.1. Participants

We had 120 volunteers from Tufts University (50 men, 70 women) aged 18 to 25 (*M* = 19.11, *SD* = 1.46) participate in the study. Participants were either compensated with course credit or $10 per hour. An a priori power analysis (1 − β = .80, α = .05, η_p_^2^ = .14) indicated that we would need a minimum of 72 participants to replicate the effect of study techniques on JOL accuracy for related items observed by Hughes et al. (2018). We randomly assigned participants to the study-practice group, the elaborative-encoding group, and the retrieval-practice group. As explained in the method section below, 21 participants could not be included in the analyses because they did not demonstrate understanding of the remember/know instructions. This drop-out rate was comparable to other remember/know studies (cf. McCabe and Geraci 2009). As a result, there were 31 participants in the study-practice group, 32 in the elaborative-encoding group, and 36 in the retrieval-practice group.

#### 2.1.2. Materials

Participants studied 44 English word pairs. Four of these word pairs were used only during the practice phase. Half of the pairs were weakly related (e.g., Throne—Castle) and the remainder were unrelated (e.g., Allergy—Divorce). Item relatedness was operationalized in terms of forward-associative strength per the University of South Florida Free Association Norms (Nelson et al. 1998). The mean forward-associative strength from prompt word to target was 2% (*SD* = 1%) for weakly related pairs, and 0% for unrelated pairs. For each word pair, we selected three words to use as foils on the four-alternative forced-choice test, which summed up to 132 foil words. Average forward-associative strength from prompt to foil was 4% (*SD* = 5%) for weakly related pairs, and 0% for unrelated pairs. All prompts, targets, and foils were nouns, ranged in length from four to eight letters, and were selected for high concreteness, which we operationalized as a value of 4 or greater on a scale ranging from 1 (highly abstract) to 7 (highly concrete) per the Nelson et al. (1998) norms. Prompts had no forward-associative strength with the targets or foils of any other pair. See Appendix A for a full list of the prompt words, targets, and foils.

#### 2.1.3. Procedure

Participants were run on computers programmed with E-Prime software (Version 2.1; Schneider et al. 2002). The procedure consisted of five phases split across two sessions: encoding of word pairs (Phases 1 and 2), 48-h retention interval (Phase 3), judgments of learning (Phase 4), and final test (Phase 5). Before each of the two experimental sessions, participants engaged in a practice session with four word-pairs. Participants were told that the tasks in the practice phase and the experiment would be identical. 

The primary purpose of the practice phase was to familiarize participants with the instructions and the tasks. The secondary purpose of the practice phase was to include a manipulation check to determine if participants understood the remember/know instructions. Following the recommendations of Yonelinas et al. (2010), we asked participants to provide explanations of their remember/know responses. Only those participants who provided answers consistent with the provided definitions were included in the subsequent analyses. 

Phase 1. Participants in all groups studied all 40 word-pairs. Participants were informed that they would see pairs of words one at a time for a short duration and that the goal was to remember these words for a later memory test. Each word pair was presented for 6000 ms. The presentation order was randomized across participants.

Phase 2. This phase varied across the three groups. As in Phase 1, Phase 2 trials were randomized across participants.

Study Practice. Participants in the study-practice group read identical instructions as those presented in Phase 1. They then saw the same 40 word-pairs, one at a time, for 6000 ms each.

Elaborative Encoding. Participants in the elaborative-encoding group were instructed that they would be studying the same word pairs a second time, but would be asked to generate single word that would thematically unite the prompt word and target of each pair together (the mediator word; e.g., “Space” for the pair “Moon—Galaxy”). Participants in the elaborative-encoding group were presented with the 40 word-pairs, one at a time, for 6000 ms each, during which time they could type their mediator word on the screen. After 6000 ms elapsed, the screen advanced to the next pair regardless of whether participants had generated their mediator word. Participants failed to generate a mediator on 6% of trials (2% of related items and 10% of unrelated items). 

Retrieval Practice. Participants in the retrieval-practice group were told that they would be taking a test on the words they studied in Phase 1, in which they would see the prompt word and be asked to provide the target. Participants in the retrieval-practice group were presented with the prompt word from all 40 word-pairs, one at a time, for 5000 ms each. While the prompt word remained on the screen, the participants were asked to provide the corresponding target. Responses were typed by the participant into the computer. After the 5000 ms elapsed, participants could no longer provide an answer, and were presented with the intact word pair for 1000 ms. Mean accuracy during retrieval practice was 48% (59% for related items and 37% for unrelated items). Participants left 22% of responses blank (15% of related items and 28% of unrelated items). 

Phase 3. This phase consisted of a 48-hr retention interval. 

Phase 4. After the retention interval, participants returned to the lab to begin Phase 4, during which participants made judgments of learning using the pre-recall and monitoring procedure (Nelson et al. 2004). Participants were presented with the prompt word from each of the 40 word-pairs, one at a time, and were asked to provide the corresponding target. Responses were typed by the participant into the computer. Participants were instructed to type the word “blank” if they could not retrieve the target. There was no time limit to respond. The average time to enter a cued-recall response was 6668 ms (*SD* = 2239 ms) in the study-practice group, 7772 ms (*SD* = 3087 ms) in the elaborative-encoding group, and 6334 ms (*SD* = 2291 ms) in the retrieval-practice group.

After entering their response for a given word pair, participants were immediately asked to make a JOL on a scale of 0–100%. Participants were instructed to estimate the probability of correctly recognizing the corresponding target on a list of four choices, without guessing, on a test that would occur in about 5 min. Participants were informed that a JOL of 0% meant that they would not be able to select the target without making a complete guess. There was no time limit to respond. The average time to enter a JOL was 2784 ms (*SD* = 983 ms) in the study-practice group, 2987 ms (*SD* = 986 ms) in the elaborative-encoding group, and 3081 ms (*SD* = 644 ms) in the retrieval-practice group.

After making their JOL for a given word pair, participants were asked to indicate whether the presented prompt word evoked a remember, know, or no memory experience. Instructions were adapted from Geraci et al. (2009). Remember responses were defined as the conscious recollection of some aspect of the original encoding experience. Participants were told to reply with a remember response only if they could provide details of what was remembered if asked by the experimenter. Know responses were defined as the feeling that a given prompt word had been encountered before during Phases 1 and 2, but did not evoke the recollection of specific information pertaining to the encoding event. A response of no memory was to be used when a given prompt word evoked neither the recollection of specific details, nor a feeling of familiarity that the prompt word had been presented during Phases 1 and 2. Participants were further instructed that remember, know, and no memory responses did not represent different levels of confidence of future recognition. To avoid interpretational ambiguity of the terms “remember” and “know,” we substituted these terms with “Type 1” and “Type 2” memory, respectively, in the instructions (see, McCabe and Geraci 2009). After participants read the remember/know instructions, the experimenter asked whether participants felt they understood the instructions, and then to explain what each response meant in their own words and with examples. If the participant did not understand or did not answer correctly, the experimenter reread the written instructions with no further elaboration. Participants were asked to provide written explanations for remember/know responses only during the practice portion of the task (four trials). Average time to enter a remember/know response was 1925 ms (*SD* = 505 ms) in the study-practice group, 2155 ms (*SD* = 756 ms) in the elaborative-encoding group, and 2342 ms (*SD* = 1133 ms) in the retrieval-practice group. For the proportions of remember, know, and no memory responses for each group, refer to Table 1.

All tasks in Phase 4 were self-paced and word pairs were presented randomly. To summarize, for each word pair, participants made a cued-recall attempt, JOL, and remember/know/no memory response before proceeding to the next word pair (see Figure 2).

Phase 5. To end the experiment, participants took a four-alternative forced-choice test. Cues from the 40 word-pairs were randomly presented one at a time. Participants were asked to select the corresponding target word from a list including three foils. There was no time limit to respond. The average time to enter a response on each final-test item was 4941 ms (*SD* = 1384 ms) in the study-practice group, 4749 ms (*SD* = 1624 ms) in the elaborative-encoding group, and 4122 ms (*SD* = 1025 ms) in the retrieval-practice group. Performance on the final test was measured as the proportion of correct responses. After each response, participants entered their confidence in their answer on a scale of *complete guess*, *low*, *medium*, and *high.* There was no time limit to respond. The average time to enter a confidence response on each test item was 1212 ms (*SD* = 283 ms) in the study-practice group, 1317 ms (*SD* = 402 ms) in the elaborative-encoding group, and 1272 ms (*SD* = 422 ms) in the retrieval-practice group.

### 2.2. Results

All analyses used an alpha rate of .05, and all post hoc pairwise comparisons were adjusted with a Bonferroni correction. As previously discussed, 21 participants were dropped from the analyses because they did not demonstrate understanding of the remember/know instructions. Dropping these participants did not affect the pattern of results or statistical significance of the tests that did not analyze the remember/know data. Additionally, although we collected data on confidence during the final test, and mean JOL magnitude, we did not include analyses on these data because they were not relevant to the central hypotheses. However, we present these data in Table 2. 

Although we used a 3 × 2 mixed design, we did not use two-way mixed ANOVA procedures to analyze the results involving gamma correlations. This is because, for many of the gamma correlations we calculated, a considerable number of participants had at least one missing cell for one of the two item types due to an incalculable correlation. For example, for JOL accuracy, 38 of the 99 participants had a gamma correlation missing for either unrelated or related items. When a participant had one gamma correlation but not the other, a 3 × 2 ANOVA would have excluded their data entirely from the analysis. Consequently, we conducted a one-way ANOVA for unrelated and related items separately, thereby preserving the maximum amount of usable data for the analyses. Follow-up *t*-tests were two-tailed and adjusted with a Bonferroni correction unless otherwise stated.

We begin our results section by examining potential differences in JOL accuracy across experimental groups and conditions. The remainder of the results section investigates factors that could explain differences in JOL accuracy between groups, including cue accessibility, cue utilization, and cue diagnosticity. Each of these factors was investigated first before conducting a planned mediation model to explore differences in JOL accuracy as a function of these three factors/potential mediators. All data can be found at https://osf.io/j9k5b/.

#### 2.2.1. Final-Test Performance

Final-test performance was measured as the proportion of correct responses on the four-alternative forced-choice test. We conducted a 3 (study technique: study practice, elaborative encoding, retrieval practice) × 2 (item type: unrelated, related) mixed-groups factorial ANOVA, which showed a main effect of study-technique group, *F*(2, 96) = 9.21, *p* < .001, η_p_^2^ = .16. Neither item type, *F*(1, 96) = 0.05, *p* = .818, η_p_^2^ < .001, nor the interaction between study-technique group and item type, *F*(2, 96) = 1.79, *p* = .173, η_p_^2^ = .04, were significant. Post hoc comparisons showed that retrieval practice (*M* = .85) led to higher final-test performance than study practice (*M* = .69), *t*(66) = 3.82, *p* < .001, *d* = 0.87, as did elaborative encoding (*M* = .84), *t*(62) = 3.66, *p* = .001, *d* = 0.86. The difference between retrieval practice and elaborative encoding was not significant, *t*(65) = 0.05, *p* > .999, *d* = 0.01. Refer to Table 2 for the means.

#### 2.2.2. JOL Accuracy

To measure JOL accuracy, we calculated intraindividual gamma correlations between JOLs and final-test performance on an item-by-item basis (Nelson 1984, 1996). These gamma correlations were calculated separately for unrelated items and related items. Due to invariance either in JOLs or final-test performance, gamma correlations for unrelated and/or related items could not be computed for some participants, which is reflected in the degrees of freedom in the subsequent analyses.

**Figure 3:**
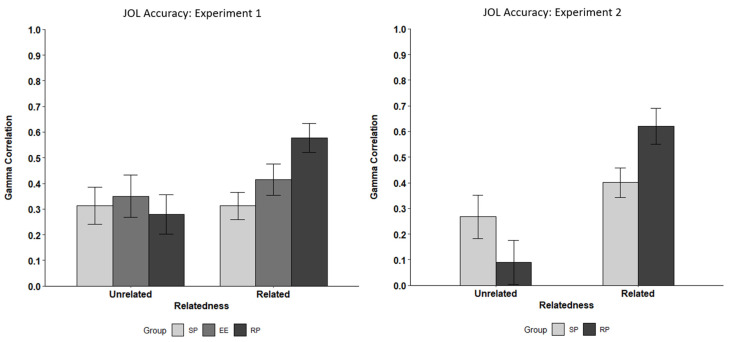
Delayed judgment-of-learning accuracy as a function of study-technique group and item type. SP = study practice; EE = elaborative encoding; RP = retrieval practice. Error bars represent the standard error of the mean.

Study techniques did not influence JOL accuracy for unrelated items, *F*(2, 75) = 0.22, *p* = .805, η_p_^2^ = .006, but did influence JOL accuracy for related items, *F*(2, 79) = 5.44, *p* = .006, η_p_^2^ = .12. For related items, pairwise comparisons demonstrated that retrieval practice (*M* = .58) led to higher JOL accuracy for than study practice (*M* = .31), *t*(52) = 3.26, *p* = .005, *d* = 0.89. Elaborative encoding (*M* = .41) did not lead to different levels of JOL accuracy for related items than retrieval practice or study practice (*p*s > .05). Overall, JOL accuracy was higher for related (*M* = .44) than unrelated items (*M* = .31), *t*(71) = 2.77, *p* = .0035, *d* = 0.33, replicating Thomas et al. (2013). See Figure 3.

**JOL Accuracy and Correct Guessing.** The proportion of trials in which participants responded correctly to a final-test question but expressed a confidence level of “complete guess” was numerically highest in the study practice group (*M* = .06), followed by retrieval practice (*M* = .03), and then elaborative encoding (*M* = .02). 

To account for the contamination of correct guessing on JOL accuracy, we recoded all items that participants answered correctly on the final test as incorrect if they expressed a confidence level of “complete guess”. JOL accuracy changed as a result of the recoding (see Table 3). As before, study techniques did not affect JOL accuracy for unrelated items, *F*(2, 78) = 0.46, *p* = .633, η_p_^2^ = .012, but did for related items, *F*(2, 80) = 4.40, *p* = .015, η_p_^2^ = .10. The pairwise comparisons showed that for related items, retrieval practice (*M* = .57) led to higher JOL accuracy than study practice (*M* = .35), *t*(52) = 2.77, *p* = .021, *d* = 0.75, but not elaborative encoding (*M* = .39), *t*(53) = 2.29, *p* = .074, *d* = 0.61. JOL accuracy remained higher for related items (*M* = .44) than unrelated items (*M* = .37), *t*(72) = 1.82, *p* = .036, *d* = 0.21. 

Conceivably, some level of correct guessing could have occurred on the trials that participants expressed a “low confidence” response for items they responded to correctly on the final test. As with correct guesses, low confidence correct responses were highest in the study practice group (*M* = .08) compared to the elaborative encoding (*M* = .05) and retrieval practice groups (*M* = .04). We therefore recoded all items responded to correctly on the final test with a confidence rating of low or complete guess as incorrect and conducted the analysis once more. Again, study techniques did not influence JOL accuracy for unrelated items, *F*(2, 80) = 0.50, *p* = .61, η_p_^2^ = .012, but did for related items, *F*(2, 85) = 3.78, *p* = .027, η_p_^2^ = .08. For related items, the same pattern held for the pairwise comparisons, as retrieval practice (*M* = .60) led to higher JOL accuracy than SP (*M* = .40), *t*(49) = 2.73, *p* = .023, *d* = 0.73, but not EE (*M* = .49), *t*(52) = 1.58, *p* = .351, *d* = 0.40. However, the overall effect of related (*M* = .50) compared to unrelated items (*M* = .42) was no longer significant, *t*(77) = 1.53, *p* = .063, *d* = 0.18.

These analyses suggest that although correctly guessing did impact JOL accuracy, differences across groups were not due to this artifact of the measurement process.

#### 2.2.3. Cue Accessibility, Utilization, and Diagnosticity

Having demonstrated an effect of study technique on JOL accuracy for related items, we turned our attention to the factors that could explain this phenomenon, including differences in cue accessibility, utilization, and diagnosticity. Differences in any of these factors could explain the difference between retrieval and study practice and be included in a mediation analysis.

The accessibility of cues was measured as the proportion of trials in which the cue was retrieved during the JOL phase. Retrieval of the target was measured as a cued-recall success during the JOL phase. Noncriterial cues were considered retrieved when participants produced a “remember” response on trials in which the target was not retrieved during the JOL phase. Proportions were calculated for unrelated and related items separately. Note that for noncriterial cues, the denominator of the proportion considers only trials in which the target was not retrieved. Refer to Table 4 for the means of cue utilization and diagnosticity.

We hypothesized that retrieval practice and elaborative encoding would result in higher accessibility of targets and noncriterial cues than study practice. We also anticipated that this effect would be greatest with related compared to unrelated word pairs.

**Cue Accessibility.** We conducted a 3 (study-technique group) × 2 (item type: unrelated, related) mixed ANOVA on the accessibility of targets. The analysis demonstrated a main effect of study-technique group, *F*(2, 96) = 5.32, *p* = .006, η_p_^2^ = .10, item type, *F*(1, 96) = 201.52, *p* < .0001, η_p_^2^ = .68, and an interaction, *F*(2, 96) = 16.84, *p* < .0001, η_p_^2^ = .26. Simple effects analysis showed that study-technique group did not affect rates of target retrieval for unrelated items, *F*(2, 96) = 0.60, *p* = .549, η_p_^2^ = .006, but did for related items, *F*(2, 96) = 11.49, *p* < .0001, η_p_^2^ = .11. Post hoc comparisons showed that for related items, the retrieval practice group (*M* = .55) outperformed the study practice group (*M* = .28), *t*(66) = 5.11, *p* < .0001, *d* = 1.25, but not the elaborative encoding group (*M* = .44), *t*(66) = 2.16, *p* = .486, *d* = 0.53. The elaborative-encoding group did not outperform the study-practice group, *t*(65) = 2.88, *p* = .071, *d* = 0.73.

A 3 × 2 mixed ANOVA demonstrated on the accessibility of noncriterial cues showed a main effect of study-technique group, *F*(2, 96) = 4.30, *p* = .016, η_p_^2^ = .082, item type, *F*(1, 96) = 17.09, *p* < .0001, η_p_^2^ = .15, and an interaction, *F*(2, 96) = 5.90, *p* = .004, η_p_^2^ = .11. Simple effects analysis showed that study-technique group did not affect rates of noncriterial recollection for unrelated items, *F*(2, 96) = 1.32, *p* = .273, η_p_^2^ = .01, but did for related items, *F*(2, 96) = 6.77, *p* = .002, η_p_^2^ = .07. Post hoc comparisons showed that elaborative encoding (*M* = .49) led to higher rates of noncriterial recollection than study practice (*M* = .28), *t*(66) = 3.55, *p* = .008, *d* = 0.90, and retrieval practice (*M* = .30), *t*(61) = 3.40, *p* = .01, *d* = 0.83. There was no difference between the retrieval and study-practice group, *t*(65) = 0.28, *p* > .999, *d* = 0.07.

**Cue Utilization.** Cue utilization was measured by calculating intraindividual gamma correlations between retrieval of a cue (either target or noncriterial cue) during the JOL phase (0 for unretrieved and 1 for retrieved) and JOLs (0–100) on an item-by-item basis. We considered a noncriterial cue as retrieved only when participants produced a “remember” response. Thus, in our coding method, a value of 1 reflects trials in which a participant produced a “remember” response, and trials with either a “know” or “no memory” response were both coded as 0. We coded in this way because we were interested in examining the conscious retrieval of noncriterial cues, which is only reflected by the “remember” response (a “know” response merely indicates familiarity).

Correlations were calculated separately for each cue and item type. Due to invariance either in the retrieval of cues (all trials or no trials) or JOLs, gamma correlations for unrelated and/or related items could not be computed for some participants, which is reflected in the degrees of freedom in the subsequent analyses. Note that for noncriterial cues, the gamma correlations were limited to trials in which the participant did not retrieve the target.

Study techniques influenced the utilization of target retrieval for unrelated items, *F*(2, 77) = 4.54, *p* = .015, η_p_^2^ = .010, and related items, *F*(2, 92) = 5.80, *p* = .004, η_p_^2^ = .11. For unrelated items, compared to elaborative encoding (*M* = .95), both retrieval practice (*M* = .99), *t*(59) = 2.62, *p* = .03, *d* = 0.67, and study practice (*M* = .99), *t*(39) = 2.47, *p* = .047, *d* = 0.73, led to greater utilization of target retrieval. For related items, the pattern partially held. Compared to elaborative encoding (*M* = .77), utilization of target retrieval was higher for retrieval practice (*M* = .92), *t*(66) = 3.28, *p* = .004, *d* = 0.80, but not study practice (*M* = .81), *t*(57) = .82, *p* > .999, *d* = 0.21. The difference between retrieval and study practice did not reach significance, *t*(61) = 2.29, *p* = .072, *d* = 0.58. Utilization of target retrieval was higher for unrelated (*M* = .98) than related items (*M* = .84), *t*(77) = 6.87, *p* < .0001, *d* = 0.78. 

The utilization of noncriterial cues did not vary across study technique groups either for unrelated, *F*(2, 79) = 0.72, *p* = .491, η_p_^2^ = .02, or related items, *F*(2, 78) = 0.20, *p* = .819, η_p_^2^ = .01. There were no differences in utilization of noncriterial cues across item type, *t*(70) = 0.17, *p* = .868, *d* = 0.02.

Critically, there were no differences in cue utilization between retrieval practice and study practice and therefore this could not explain differences in JOL accuracy between these groups. 

**Cue Diagnosticity.** To measure the degree to which cues were diagnostic of final-test performance, we calculated gamma correlations between retrieval of each cue (target or noncriterial) during the JOL phase (coded as 0 for unretrieved or 1 for retrieved) and final-test performance (coded as 0 for incorrect and 1 for correct) on an item-by-item basis for each participant. Correlations were calculated separately for each cue and item type, yielding four correlations for each participant. As with utilization, for noncriterial cues, the gamma correlations were limited to trials in which the participant did not retrieve the target.

Because both variables in the gamma correlations were binary, we applied the Snodgrass and Corwin (1988) correction, which is used extensively in signal-detection research. The correction has been adopted by researchers examining metacognitive questions concerning confidence (Higham et al. 2009), JOLs (Grainger et al. 2016; McCabe and Soderstrom 2011), and feeling-of-knowing judgments (cf. Cosentino et al. 2016; Souchay et al. 2000, 2007; Hanczakowski et al. 2013; Hébert and Peretz 1997). Calculating gamma correlations with two binary variables involves using data from a 2 × 2 contingency table and yields the same outcomes as signal-detection analyses (Swets 1986). In signal-detection terms, the cells of our 2 × 2 table represent hits (retrieved the cue and the final-test question was correct), false alarms (retrieved the cue but the final-test question was incorrect), correct rejections (did not retrieve the cue and the final-test question was incorrect), and misses (did not retrieve the cue and the final-test question was correct). The formula for gamma in this special case^1^ is (hits × correct rejections) − (false alarms × misses)/(hits × correct rejections) + (false alarms × misses). The Snodgrass-Corwin correction was designed to handle computability issues arising from cases in which two or more of the four frequencies are equal to 0, which can render measures of discrimination (like d’ or gamma) incomputable because the denominator becomes 0. Thus, Snodgrass and Corwin (1988) recommended adding .5 to each of the four frequencies and dividing this adjusted value by the number of trials + 1 before calculating measures of discrimination. For a discussion on the theoretical and statistical appropriateness of this correction, see Barrett et al. (2013). 

The diagnosticity of target retrieval did not vary across study technique groups either for unrelated, *F*(2, 96) = 1.34, *p* = .266, η_p_^2^ = .03, or related items, *F*(2, 96) = 0.87, *p* = .422, η_p_^2^ = .02. However, the diagnosticity of target retrieval was higher for related (*M* = .55) than unrelated items (*M* = .16), *t*(98) = 9.30, *p* < .0001, *d* = 0.93. A one-sample *t*-test showed that the diagnosticity of target retrieval was greater than 0 for unrelated, *t*(98) = 4.25, *p* < .0001, *d* = 0.43, and related items, *t*(98) = 18.07, *p* < .0001, *d* = 1.82.

As with target retrieval, the diagnosticity of noncriterial cues did not vary across groups, both for unrelated items, *F*(2, 96) = 1.08, *p* = .342, η_p_^2^ = .022, and related items, *F*(2, 96) = 2.40, *p* = .096, η_p_^2^ = .05. However, the diagnosticity of noncriterial cues did not vary across item type, *t*(98) = 0.30, *p* = .763, *d* = 0.03. A one-sample *t*-test showed that the diagnosticity of target retrieval was not greater than 0 for unrelated, *t*(98) = −0.97, *p* = .332, *d* = −0.98, or related items, *t*(98) = −0.55, *p* = .585, *d* = −0.06.

Importantly, there were no differences in cue diagnosticity between retrieval practice and study practice and therefore this could not explain differences in JOL accuracy between these groups.

#### 2.2.4. Cue Accessibility and JOL Accuracy

Before conducting our mediation model to explore the factors underlying group differences in delayed JOL accuracy, we examined our hypothesis that there would be a linear, positive association between cue accessibility and delayed-JOL accuracy (our potential mediators). we calculated Pearson correlations (*r*) between these two measures (a proportion and gamma correlation, respectively). There was a positive association between the accessibility of targets and JOL accuracy for related, *r*(80) = .50, *p* < .0001, and unrelated items, *r*(76) = .22, *p* = .027. 

In contrast to the accessibility of targets, there was no significant association between the accessibility of noncriterial cues and JOLs for either item type in either group (*p*s > .05). Thus, there was only a positive correlation between the accessibility of targets and delayed-JOL accuracy and not the accessibility of noncriterial cues.

#### 2.2.5. Mediation Analysis of JOL Accuracy

Having demonstrated that retrieval practice leads to higher JOL accuracy than study practice for related items, we conducted a mediation analysis to determine the cause of this effect. We only found differences between these two groups in cue accessibility and not in cue utilization or cue diagnosticity. Thus, we tested the hypothesis that study techniques influenced JOL accuracy indirectly through affecting the accessibility of (a) targets and/or (b) noncriterial cues, which served as the two mediators in the subsequent analysis (both were measured as proportions in the manner described earlier). We conducted the mediation analysis using ordinary-least-squares path analysis using the PROCESS macro (Hayes 2013) in R. Note that each relationship expressed in the model corresponds to an unstandardized coefficient from the ordinary-least-squares regression path analysis. Retrieval practice was dummy coded as 1, meaning that each coefficient pertaining to study techniques represents the difference between retrieval practice compared to study practice (coded as 0). The accessibility of targets and noncriterial cues were centered at the grand mean of the two groups. Refer to Figure 1 for a diagram of the final mediation model we tested (see Table 5 for coefficients of each of the preliminary regression models in mediation analysis). For this analysis, we did not adjust JOL accuracy for correct guessing because the pattern of significance did not change across levels of correct guessing adjustment (those analyses are presented in the Data Availability Statement).

As shown in Table 5, retrieval practice led to higher accessibility of targets than study practice, *a*_1_ = .29, *t* = 5.40, *p* < .001, and higher accessibility of targets was positively and significantly related to JOL accuracy for related items, b_1_ = .58, *t* = 3.06, *p* = .004. Retrieval practice did not lead to higher accessibility of noncriterial cues than study practice, *a*_2_ = .03, *t* = 0.13, *p* = .684, and higher accessibility of noncriterial cues was not significantly associated with JOL accuracy for related items, *b*_2_ = −.02, *t* = −0.11, *p* = .917. We tested the significance of the indirect effects of study technique on JOL accuracy for related items through the accessibility of targets (*ab*_1_ = .17) and noncriterial cues (*ab*_2_ < .001) by estimating 95% confidence intervals (CI) with bias-corrected bootstrap samples from 5000 simulations. The 95% CI for the indirect effect of the accessibility of targets was entirely above 0 [.08, .26], but the 95% CI for the indirect effect of accessibility of noncriterial cues included 0 [−.02, .03]. The direct effect of retrieval practice on JOL accuracy for related items was not significant, *c’* = .10, *t* = 1.06, *p* = .295. Therefore, the analysis suggests that the benefit of retrieval practice, over study practice, on JOL accuracy for related items was entirely mediated by its influence on the accessibility of targets during the JOL phase. 

### 2.3. Discussion

We found that study techniques influenced JOL accuracy for related, but not unrelated, items. For related items, retrieval practice, but not elaborative encoding, led to higher JOL accuracy than study practice. The results suggest that differences between groups were not the result of the contamination of correct guessing. Our findings are consistent with the hypothesis that differences between JOL accuracy across groups would be greatest with related compared to unrelated items.

The difference in JOL accuracy for related items between the retrieval and study-practice groups was not driven by differences in the utilization or diagnosticity of retrieval cues. Rather, it was the total number of targets retrieved across trials that drove the difference. The number of retrieved targets was positively correlated with JOL accuracy, and the number of retrieved targets was higher in the retrieval-practice group. A mediation analysis demonstrated that the effect of study technique on JOL accuracy was entirely mediated by the accessibility of targets. 

Our mediation analysis showed that retrieval practice led to higher JOL accuracy than study practice by increasing the accessibility of targets, but not noncriterial cues. That is, participants who learned via retrieval practice had access to more information that was diagnostic of final-memory performance than study practice, leading to better prediction accuracy. These results also suggest that study techniques did not lead to differences in JOL accuracy for unrelated items because the accessibility of targets for these items was statistically equivalent across groups.

For related items, we found that elaborative encoding led to higher accessibility of targets and noncriterial cues than study practice. However, elaborative encoding only led to numerically, and not statistically, higher levels of JOL accuracy than study practice. This contradicts the finding reported by Hughes et al. (2018). It is possible that we did not replicate the effect because, contrary to Hughes et al. (2018), we ensured that encoding time per item was equal across groups. That is, in the previous study, participants spent more time encoding items in the elaborative-encoding group (self-paced task) relative to the study practice group (fixed-pace task). By ensuring equal encoding time across groups, we likely reduced differences between these groups that would influence JOL accuracy, such as cue accessibility. 

Notably, noncriterial cues were not diagnostic of final memory performance. This finding is inconsistent with the previous literature on the subject. It is possible that only a subset of noncriterial cues is diagnostic of final-memory performance, and by casting such a broad net with the remember/know paradigm, our measure mixed diagnostic and nondiagnostic cues.

## 3. Experiment 2

The purpose of Experiment 2 was to replicate the main results of the previous experiment. We decided only to include the two study-technique groups that yielded the most important effects of interest: study practice and retrieval practice. 

Due to the COVID-19 pandemic, in-person data collection was no longer feasible. We therefore recruited participants via the Amazon Mechanical Turk platform. We created and conducted our online experiment via the Gorilla builder and platform (www.gorilla.sc; Anwyl-Irvine et al. 2020). We used the Gorilla platform because it can tightly control the duration of presented stimuli across browsers and devices. It is also precise enough to measure response time at a high level of accuracy. 

We made several design changes to accommodate the online nature of the experiment. First, piloting results made it clear that the strict standard for the retention interval between the first and second session was not practical, as few participants returned for the second session when it could only be completed exactly 48 h following completion of the first session. We therefore allowed participants to return any time between 48 and 72 h after completing their first session. Second, the entirely passive nature of the study-practice encoding task made it difficult to assess whether any participant was paying attention, or even in the same room as their computer, during the initial learning phase. We therefore added an active component to the initial learning phase to monitor engagement. Specifically, we had participants judge the semantic relatedness of each word pair after each item. We only invited participants to participate in the second session if they responded to these prompts accurately on 70% of trials, thereby ensuring roughly equal task engagement across the two encoding conditions. Third, the procedure for teaching participants about the distinction between remember, know, and no-memory responses could no longer rely on live, verbal interaction with the research assistant. We therefore adopted an iterative, quiz-based procedure that would not let participants proceed to the next phase of the experiment until they could answer all questions correctly.

### 3.1. Materials and Methods

#### 3.1.1. Participants

Power Analysis. An a priori power analysis demonstrated that to replicate the effect of retrieval practice relative to restudy on JOL accuracy, a total of 40 participants would be needed (1 − β = .80, α = .05, *d* = .93). We conducted an additional a priori power analysis to determine the sample size needed to replicate the mediation model from Experiment 1 (1 − β = .80, α = .05) using a Montecarlo-simulation approach with 1000 replications (Schoemann et al. 2017). This analysis demonstrated that we would need a total of 58 participants to achieve the desired level of power. We anticipated a relatively high rate of participant exclusion and attrition rate by using the Amazon Mechanical Turk platform and therefore recruited more participants than would be needed to achieve the minimum sample-size requirements. Participants were compensated $7.00 for completing the experiment.

Recruitment Criteria. Participants were only allowed to join the study if they used a laptop or desktop computer, were between the ages of 18 and 25, were in the United States, and had a 97% completion rate for surveys/studies on Amazon Mechanical Turk.

Final Sample. We recruited a total of 206 participants through the Amazon Mechanical Turk platform and randomly assigned them to one of the two study-technique groups. Before data collection, we set a performance criterion for participants to achieve during the initial learning phase to qualify for the second session of the experiment, which was a 70% accuracy rate in judging word pairs as related or unrelated. A total of 177 participants achieved or exceeded this threshold and were invited for the second session, reducing the sample size by 14%. Of these participants, 86 returned and completed the study, representing an additional 51% loss in sample size. Ultimately, there were 86 participants (*M_age_* = 23.64, *SD* = 1.93) with 43 participants in each study-technique group. Of the final sample, 41 identified as women, 43 as men, and 2 as neither.

#### 3.1.2. Materials

We used the same word pairs as Experiment 1. Due to a coding error, two of the unrelated pairs were not presented during the final test.

#### 3.1.3. Procedure

Participants completed the experiment online via the Gorilla online platform. The procedure was identical to that of Experiment 1 except for the following changes noted below. 

Phases 1 and 2: Initial Encoding. We did not include the elaborative-encoding group. We also now had participants rate the degree of semantic relatedness of words in each pair. After studying, the pair disappeared, and a prompt asked the participant to assess the relatedness of the two words by clicking a button labeled unrelated or related. The screen advanced after 2500 ms regardless of whether participants responded. Participants responded to the related/unrelated judgment accurately 92% of the time (*SD* = 7%). In the retrieval-practice group, participants recalled the target on 46% of trials (*SD* = 19%). Recall success was 67% (*SD* = 20%) for related items and 24% (*SD* = 23%) for unrelated items.

Phase 3: Retention Interval. Third, the duration of the retention interval was not fixed to 48 h, but ranged from 48 h to 72 h (*M* = 57.44, *SD* = 9.65).

Phases 4 and 5: JOL and Final Test. These phases were identical except for two changes. First, participants expressed their responses by clicking buttons rather than typing their responses. For JOLs, there were 10 buttons, arranged from left to right, in increments of 10% with the same verbal anchors as Experiment 1. For final test and confidence responses, there was a grid of four buttons that participants could select. Second, we changed the way that we tested participants’ knowledge of the distinction between remember, know, and no memory responses. We used the same instructions as Experiment 1 but changed the procedure in which we reinforced this knowledge and verified that participants sufficiently understood the distinction to proceed to the next parts of the experiment. To ensure participants adequately understood the instructions, we used a quiz-based procedure to test and reinforce their knowledge of the topic. After reading the instructions, participants took a quiz that consisted of 11 three-alternative-forced choice questions. The questions involved pairing definitions with the appropriate memory response and having participants read short vignettes about a fictional participant’s memorial experiences with a word pair and indicating the types of responses they should express. Explanatory feedback was provided after each correct response. For questions that were answered incorrectly, the feedback merely consisted of a statement that the response was incorrect. Rather than excluding participants based on their response accuracy, we did not permit participants to proceed to the next part of the experiment until they correctly answered all questions. We used the number of responses it took for participants to achieve a perfect score on the quiz as an index of how well they understood the content (a perfect score on the first try would be 11 responses). On average, it took participants 12.79 responses to achieve a perfect score on the quiz (*SD* = 1.74).

During the JOL phase, the average response time in the study practice group was 1708 ms (*SD* = 651 ms) and in the retrieval practice group it was 1578 ms (*SD* = 614 ms). When making the remember/know judgments, participants averaged a response time of 1388 ms (*SD* = 496 ms) in the study practice group, and 1271 ms (*SD* = 454 ms) in the retrieval practice group. On the final test, it took an average of 3861 ms (*SD* = 1274 ms) to respond in the study practice group, and 2922 ms (*SD* = 1064 ms) in the retrieval practice group. Finally, for the confidence judgments, response time in the study practice group took was 932 ms (*SD* = 222 ms), and it was 825 ms (*SD* = 232 ms). 

### 3.2. Results

As with Experiment 1, we did not conduct the 2 (study-technique group: study practice, retrieval practice) × 2(item type: unrelated, related) mixed ANOVA for the analyses involving gamma correlations. *T*-tests were two-tailed unless otherwise stated. Follow-up *t*-tests were adjusted with a Bonferroni correction. All data can be found at https://osf.io/j9k5b/.

#### 3.2.1. Retention Interval

The duration of the retention interval did not differ between the study-practice (*M* = 57.55 h) and retrieval-practice groups (*M* = 57.33 h), *t*(84) = 0.10, *p* = .918, *d* = .02. Furthermore, the length of the retention interval did not significantly correlate with any of the dependent measures of interest, including JOL accuracy, the accessibility of targets/noncriterial cues, remember/know/no memory responses, and final-test accuracy (all *p*s > .05).

#### 3.2.2. Final-Test Performance

A 2 (study-technique group: study practice, retrieval practice) × 2 (item type: unrelated, related) mixed ANOVA demonstrated a main effect of study-technique group *F*(1, 84) = 8.71, *p* = .004, η_p_^2^ = .09. Retrieval practice (*M* = .81) led to higher final-test scores than study practice (*M* = .69). There was also a main effect of item type, with performance being higher for related (*M* = .81) than unrelated items (*M* = .69), *F*(1, 84) = 45.78, *p* < .0001, η_p_^2^ = .35. The interaction was not significant, *F*(1, 84) = 3.15, *p* = .078, η_p_^2^ = .036. Refer to Table 6 for the means.

#### 3.2.3. JOL Accuracy

For unrelated items, JOL accuracy was numerically, but not statistically, different between retrieval (*M* = .09) and study practice (*M* = .26), *t*(71) = 1.40, *p* = .165, *d* = 0.33. However, there was a difference for related items, as retrieval practice (*M* = .62) led to higher JOL accuracy than study practice (*M* = .44), *t*(63) = 2.09, *p* = .021, *d* = 0.53 (one-tailed). Overall, JOL accuracy was higher for related (*M* = .51) than unrelated items (*M* = .18), *t*(57) = 4.08, *p* = .0001, *d* = 0.54 (one-tailed).

JOL Accuracy and Correct Guessing. Rates of correct guessing were close, but numerically slightly higher, for the study practice (*M* = .06) compared to the retrieval practice group (*M* = .04). Nevertheless, any rate of correct guessing can add noise to the measurement of JOL accuracy. For completeness and to mirror the analyses of Experiment 1, we therefore recoded all correct guesses as incorrect responses on the final test. As before, for unrelated items, there was no statistical difference between retrieval practice (*M* = .06) and study practice (*M* = .25), *t*(69) = 1.36, *p* = .161, *d* = 0.38. For related items, the effect remained significant. Retrieval practice (*M* = .64) led to higher JOL accuracy than study practice (*M* = .49), *t*(67) = 1.69, *p* = .048, *d* = 0.41 (one-tailed). Further, JOL accuracy remained higher for related (*M* = .55) compared to unrelated items (*M* = .16), *t*(60) = 5.26, *p* < .0001, *d* = 0.67 (one-tailed). 

We then recoded all low confidence responses as incorrect on the final test. Rates of correct low responses were nearly identical between the retrieval practice (*M* = .104) and the study practice group (*M* = .097). This time, there was no significant difference across groups for either unrelated, *t*(75) = 0.83, *p* = .401, *d* = 0.19, or related items, *t*(76) = 1.14, *p* = .129, *d* = 0.26 (one-tailed). Nevertheless, JOL accuracy remained considerably higher for related (*M* = .60) than unrelated (*M* = .17) items, *t*(70) = 6.58, *p* < .0001, *d* = 0.78 (one-tailed). Refer to Table 7 for the means across levels of correct guessing adjustments.

#### 3.2.4. Cue Accessibility, Utilization, and Diagnosticity

Values were calculated in the same manner as Experiment 1. 

**Cue Accessibility.** We conducted a 2 × 2 ANOVA on the accessibility of targets. There was a main effect of study-technique group, *F*(1, 84) = 13.80, *p* = .0004, η_p_^2^ = .14. Retrieval practice (*M* = .35) outperformed study practice (*M* = .19). There was also a main effect of item type, *F*(1, 84) = 236.57, *p* < .0001, η_p_^2^ = .74. Performance for related items (*M* = .45) was higher than unrelated items (*M* = .11). There was also an interaction, *F*(1, 84) = 16.69, *p* = .0001, η_p_^2^ = .17. Follow-up simple effects analysis showed that for unrelated items, the difference between groups barely missed significance, *F*(1, 84) = 3.58, *p* = .06, η_p_^2^ = .041. However, the difference was significant for related items, *F*(1, 84) = 19.24, *p* < .0001, η_p_^2^ = .19. Refer to Table 7 for the means.

For the accessibility of noncriterial cues, a 2 × 2 ANOVA showed no main effect of group, *F*(1, 83) = 0.49, *p* = .487, η_p_^2^ = .01. The main effect of item type was significant, *F*(1, 83) = 36.27, *p* < .0001, η_p_^2^ = .30. Accessibility of noncriterial cues was higher for related items (*M* = .13) compared to unrelated items (*M* = .005). The interaction was also significant, *F*(1, 83) = 6.93, *p* = .010, η_p_^2^ = .08. However, the follow-up simple-effects analysis demonstrated no effect of study-technique group for unrelated items, *F*(1, 84) = 0.13, *p* = .722, η_p_^2^ < .01, or related items, *F*(1, 83) = 2.15, *p* = .146, η_p_^2^ = .03.

**Cue Utilization.** Utilization of targets (*M* = .87) did not vary across groups for unrelated items, *t*(40) = 0.47, *p* = .644, *d* = 0.15, or related items, *t*(76) = 0.44, *p* = .666, *d* = 0.10. Utilization of target retrieval did not vary across unrelated and related items, *t*(40) = 1.73, *p* = .091, *d* = 0.27. Refer to Table 8 for the means.

The utilization of noncriterial cues (*M* = .83) did not vary across groups for unrelated items, *t*(43) = 0.73, *p* = .472, *d* = 0.22, or related items, *t*(58) = 1.18, *p* = .242, *d* = 0.31. Utilization of noncriterial cues did not vary across unrelated and related items, *t*(37) = 0.33, *p* = .74, *d* = 0.05. 

**Cue Diagnosticity.** The diagnosticity of target retrieval (*M* = .26) did not vary across groups for either unrelated items, *t*(84) = 0.44, *p* = .661, *d* = 0.10, or related items, *t*(84) = 0.45, *p* = .654, *d* = 0.10. However, the diagnosticity of target retrieval was higher for related (*M* = .50) compared to unrelated (*M* = .02) items, *t*(85) = 9.44, *p* < .0001, *d* = 1.02. Refer to Table 8 for the means.

For unrelated items, the diagnosticity of noncriterial cues was higher for study practice (*M* = −.04) compared to retrieval practice (*M* = −.33), *t*(84) = 2.91, *p* = .005, *d* = 0.63. However, the difference for related items between study practice (*M* = −.05) and retrieval practice (*M* = −.22) did not reach significance, *t*(84) = 1.86, *p* = .067, *d* = 0.40. Collapsed across groups, there was no difference in diagnosticity between unrelated and related items, *t*(85) = 0.87, *p* = .388, *d* = 0.09. 

#### 3.2.5. Cue Accessibility and JOL Accuracy

Before conducting the mediation analysis, we examined the association between the accessibility of targets and noncriterial cues and JOL accuracy. There was a positive association between the accessibility of targets and JOL accuracy for unrelated, *r*(71) = .43, *p* = .0002, and related items, *r*(63) = .25, *p* = .023. However, the association between the accessibility of noncriterial cues and JOL accuracy was only significant for unrelated items, *r*(71) = .29, *p* = .014, and not for related items, *r*(63) = .08, *p* = .549.

#### 3.2.6. Mediation Analysis of JOL Accuracy

We conducted a mediation analysis in the same manner as Experiment 1, including both mediators—(1) the accessibility of targets and (2) noncriterial cues. For this analysis, we did not adjust JOL accuracy for correct guessing because the pattern of significance did not change across levels of correct guessing adjustment (those analyses are presented in the Data Availability Statement).

For related items, retrieval practice led to higher accessibility of targets than study practice, *a*_1_ = .14, *t* = 7.59, *p* < .0001. However, in the final model, higher accessibility of targets was only numerically, but not significantly, associated with JOL accuracy for related items, *b*_1_ = .14, *t* = 1.44, *p* = .156. Consequently, in contrast to Experiment 1, this indirect pathway was not significant, *ab*_1_ = .049 (95% CI [−.02, .13]). The indirect pathway involving noncriterial cues was also not significant, *ab*_2_ = −.004 (95% CI [−.04, .02]). After inclusion of these mediator pathways, the direct effect of study-technique group on JOL accuracy was no longer significant, *c’* = .14, *t* = 1.47, *p* = .146. Refer to Table 9 for each model in the path analysis.

### 3.3. Discussion

The results of Experiment 2 partially replicated the findings of the prior experiment. For related, but not unrelated items, retrieval practice led to higher JOL accuracy than study practice. However, the effect size was smaller than Experiment 1 (*d* = .93 vs. *d* = .53). The effect was reduced, but remained statistically significant, even when recoding correct guesses on the final test as incorrect, but not when taking a further step and recoding all low confidence responses as incorrect. The analyses therefore suggested that some of the group differences in JOL accuracy may have been attributable to the contamination of correct guessing. 

As with Experiment 1, we found that for related items, retrieval practice enhanced the rate of target retrieval, and higher rates of target retrieval were positively associated with JOL accuracy. However, the mediation analysis examining this indirect pathway did not achieve statistical significance. Note, though, that the analysis was partially consistent with Experiment 1. After the inclusion of the accessibility of target retrieval was entered into the model, the effect of study technique was no longer significant. The results therefore trended in the same way as Experiment 1 but did not achieve significance. 

We also found that JOL accuracy was higher for related than unrelated items, an effect that did not diminish across both of the two corrections for correct guessing. This makes sense because target retrieval was substantially higher for related than unrelated items. Altogether, the results of Experiment 2 indicate that higher levels of target recall are positively associated with JOL accuracy.

## 4. Meta-Analysis of Experiments 1 and 2

Due to the discrepancy in results between the two experiments, we took a meta-analytic approach to determine if the effects of interest were significant when pooling across the datasets. We first examined JOL accuracy across groups and then turned our attention to the mediation models. 

Note that an important decision in meta-analytic procedures is whether to use fixed-effects or random-effects models. This decision hinges, in part, on assessing the heterogeneity of the effect sizes. This is commonly assessed via a Cochran’s Q test, in which a significant result indicates that there is heterogeneity, and thus a random-effects model should be used (Kanters 2022). However, the power of the Cochran’s Q test is low when the number of studies or experiments included in the analyses is low (Hardy and Thompson 1998). Consequently, some researchers advocate for the use of a less conservative criterion to assess the significance of the Cochran Q test (α = .10; Dickersin and Berlin 1992). In all of the following meta-analytic tests, the *p*-value of Cochran Q tests ranged from .17 to .47. A complementary way of assessing heterogeneity is not just to examine the significance of the Cochran’s Q test, but to look at the degree of heterogeneity via the *i*^2^ statistic, which quantifies heterogeneity as a percentage. In all but one case, *i*^2^ was low per the cutoffs of Higgins et al. (2019; ≤30%). In the remaining case, it was medium per those cutoffs (47%). A fixed-effects approach is also normally preferable when the primary aim of the analysis is to draw conclusions about a set of similar experiments within a study as opposed to generalizing more broadly, as a random-effects approach tends to be too conservative for that aim (Goh et al. 2016). Given the conjunction of the non-significant Cochran’s Q tests, the low-to-medium *i*^2^ values, and the highly similar design and sample sizes of Experiments 1 and 2, we adopted a fixed-effects approach. However, a word of caution is in order because our use of a fixed-effects approach means that the results would be less likely to generalize to less similar experiments and methodologies, especially because we only have two experiments in the sample. 

### 4.1. JOL Accuracy

**Figure 4:**
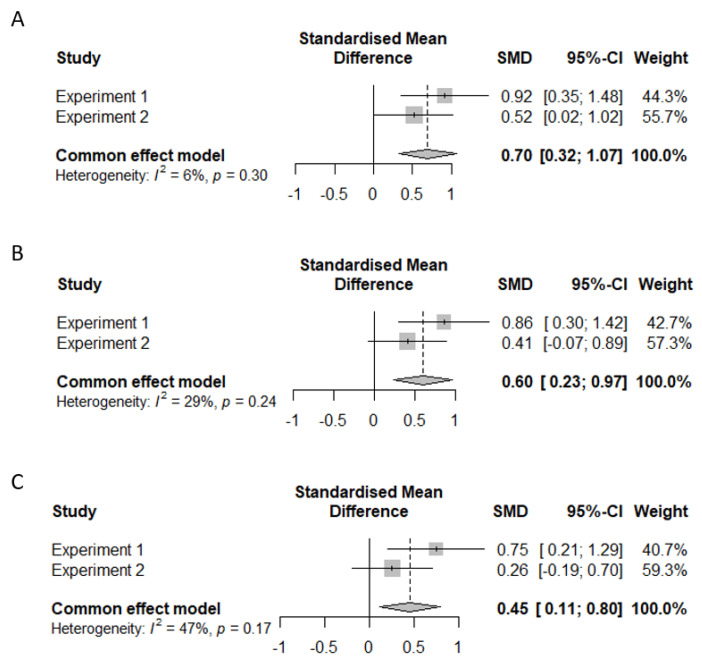
Meta-analysis of JOL accuracy for related items. (**A**) Effect sizes without adjustment for correct guessing. (**B**) Effect sizes with adjustment of correct guessing. (**C**) Effect sizes with adjustment for correct guessing and low confidence. Effect sizes are Hedges’ g.

In Experiment 1, JOL accuracy was higher for retrieval compared to study practice, even when accounting for levels of correct guessing in two ways. However, in Experiment 2, the differences in JOL accuracy across groups did not entirely persist across the analyses accounting for correct guessing. We therefore conducted a miniature meta-analysis of these results to determine the reliability of these effects using the Meta package (Balduzzi et al. 2019) in R (R Core Team 2022) using a fixed-effects modeling approach. The effect size was the standardized-mean difference with Hedges’ adjustment (Hedges’ g; Hedges 1981). As shown in Figure 4, JOL accuracy for related items was significant across adjustments for correct guessing and/or low confidence correct responses on the final test. The analysis code and output can be found in the Data Availability Statement. 

### 4.2. Mediation Analysis of JOL Accuracy

We also conducted a meta-analysis of the mediation models, since the significance of the indirect pathway of study-technique group → target accessibility → JOL accuracy (related items) was significant in Experiment 1, but not 2. We used a two-stage meta-analytic structural equation modeling (TSSEM) approach on the direct and indirect effects of our mediation models (Cheung and Chan 2005). The two-stage approach has multiple advantages relative to single-stage approaches (see, Viswesvaran and Ones 1995). We conducted our analyses with the METASEM package (Cheung 2015) and OpenMX (Boker et al. 2011) in R. The analysis code and output can be found in the Data Availability Statement. 

The first stage of the TSSEM uses a fixed-effects, multivariate meta-analytic model to create a pooled, sample-size weighted correlation matrix. Our pooled matrix included the independent variable (study-technique group), the two mediators (target and noncriterial cue accessibility), and the dependent variable (JOL accuracy of related items). Pooled across experiments, the positive association between retrieval practice and JOL accuracy was significant, *r* = .34, *p* < .0001. There were also positive associations between retrieval practice and the accessibility of targets, *r* = .46, *p* < .0001, and the accessibility of targets and JOL accuracy, *r* = .40, *p* < .0001. None of the other correlations were significant (*p*s > .05). Parameters of the Stage-1 pooled correlation matrix is shown in Table 10.

Before proceeding to the second stage of the analysis, it was necessary to evaluate the homogeneity of effect-size variance. If the effect-size variance is too heterogenous, then a random-effects model should be used in Stage 2. A Cochran’s *Q* test rejected the alternative hypothesis that the effect sizes were heterogenous, $\chi^{2}$ (*df* = 6, N = 119) = 5.60, *p* = .469. Corroborating the *Q* statistic, the degree of heterogeneity was low and negative (*i*^2^ = −7%) and therefore equivalent to 0 (Higgins et al. 2019). Consequently, a fixed-effects model was appropriate for the second stage of the analysis.

The second stage of the TSSEM involved using weighted least squares to fit a structural equation model to the pooled correlation matrix as though it were the observed data. We specified a mediation model in the same manner as Experiments 1 and 2. The results of the Stage-2 model are depicted in Table 11. Retrieval practice increased the accessibility of targets (β = .46, 95% CI [.32, .60]), and rates of target retrieval were positively associated with JOL accuracy of related items (β = .30, 95% CI [.12, .49]). Overall, this indirect pathway was significant, (β = .14, 95% CI [.06, .26]). None of the paths involving noncriterial cues were significant, including the indirect pathway (β = .002, 95% CI [−.02, .03]). Contrary to Experiment 1, this model did not yield complete mediation, as the direct effect of retrieval practice on JOL accuracy was still significant, (β = .20, 95% CI [.01, .39]). Nevertheless, the effect size of study technique on JOL accuracy decreased from the Stage 1 (β = .34) to the Stage 2 (β = .20) model, indicating partial mediation (Baron and Kenny 1986).

## 5. General Discussion

In two experiments, we found that study techniques influenced JOL accuracy for related, but not unrelated, items. For related items, retrieval practice, but not elaborative encoding, led to higher JOL accuracy than study practice. This result is consistent with the hypothesis that differences between JOL accuracy across groups would be greatest with weakly related compared to unrelated items. To explain differences in JOL accuracy across groups, we explored the properties of the underlying cues that participants used to make their JOLs. We found that although participants used target retrieval and noncriterial cues to make their JOLs, only target retrieval was diagnostic of final-memory performance. 

We examined the hypothesis that study techniques impact delayed JOL accuracy by affecting the quantity of diagnostic retrieval cues stored in long-term memory. Mediation analyses with the study and retrieval practice groups support this account. For related items in both experiments, retrieval practice enhanced the number of targets retrieved at the time of the JOL, and the rate of target retrieval was positively associated with JOL accuracy. Although this indirect pathway was only significant in Experiment 1, a meta-analysis showed that this indirect pathway was significant when pooling across both experiments. Of note, for unrelated items, the accessibility of targets was also positively associated with JOL accuracy in both experiments. However, in both experiments, retrieval practice did not enhance the accessibility of unrelated targets relative to study practice, which explains why it also did not enhance JOL accuracy for those items.

### 5.1. Target Retrieval and JOL Accuracy

The results of this study also speak to the factors underlying delayed-JOL accuracy. We demonstrated that delayed-JOL accuracy can depend on the number of correct targets retrieved during the prediction phase, which contradicts earlier proposals that relative JOL accuracy should be logically independent of absolute memory performance (Nelson 1984; Gonzalez and Nelson 1996). This effect likely owes to the fact that the higher the number of retrieved targets, the fewer the number of trials in which participants must base their JOLs on the retrieval of non-diagnostic cues or no cues at all. When participants retrieve a non-diagnostic cue, they might express a high JOL but nevertheless get that item wrong on the four-alternative forced-choice test. When participants do not retrieve a cue, they might express a low JOL but still recognize that target on the four-alternative forced-choice test”). Both types of trials would induce mismatches between JOLs and final-test performance, reducing prediction accuracy. Increasing the number of targets retrieved at the time of the JOL reduces the potential number of these problematic cases, preserving JOL accuracy.

This explanation also accounts for our finding that, in both experiments, retrieval of the target was more diagnostic for related items (*M* = .54 and *M* = .50, respectively) compared to unrelated items (*M* = .16 and *M* = .02, respectively). The comparatively higher diagnosticity for related items likely owes to the discrepancy in the performance of target recall and recognition. In Experiment 1, participants retrieved 44% of related targets and 22% of unrelated targets during the cued-recall portion of the JOL phase. However, on the four-alternative forced-choice test, participants recognized 79% of weakly related and 79% of unrelated targets. The discrepancy between the recall and recognition of related items (+35%) was smaller than unrelated items (+57%). In Experiment 2, these discrepancies were nearly identical for related and unrelated items (+36% and +58%, respectively). This means that for both item types, there were many instances in which participants failed to retrieve the target, but ultimately recognized it on the four-alternative forced-choice. Such cases reduce the diagnosticity of target retrieval as a monitoring cue. However, there were more of these cases for unrelated items, resulting in comparatively lower diagnosticity of target retrieval and therefore, lower JOL accuracy.

### 5.2. Cued-Recall Final Tests

Most JOL studies use cued-recall rather than recognition tests as the final measure of memory (for a review see Rhodes and Tauber 2011a), and thus it is worth considering how our findings would extend to such designs. One reason our findings may be attenuated in these designs concerns how the diagnosticity of target retrieval changes as a function of final-test type. Target retrieval, which is measured via cued-recall, is much more diagnostic when the final test is also cued recall rather than recognition (e.g., Hertzog et al. 2013). The higher the diagnosticity of target retrieval, the lower the probability of the metacognitive errors we described earlier (e.g., failed target retrieval with a low JOL, but later memory success on the final test). In our study, we argued that increasing the number of retrieved targets benefitted JOL accuracy by decreasing the number of metacognitive errors that can occur when the target is not retrieved. However, in designs using a final test that is cued-recall rather than recognition, there would likely be fewer of these metacognitive errors. Because there would be fewer of these metacognitive errors to reduce, the positive influence of the number of retrieved targets on JOL accuracy would be attenuated. Consequently, the influence of study techniques on JOL accuracy might also be reduced, since this effect owes to how these techniques influence the number of targets retrieved.

However, it is conceivable that our results would extend to experiments with a final cued-recall test when the retention interval is sufficiently large. Consider that in a usual JOL paradigm, the retention interval between JOLs and final-test is quite short—no more than 30 min in almost every study that reports the duration of this interval (for a review, see Rhodes and Tauber 2011a). The short retention interval means that targets retrieved at the prediction phase are highly likely to be remembered again on the final test. Consequently, the cue of target retrieval is highly diagnostic in this paradigm. Increasing the retention interval between JOL and the final test would reduce the likelihood of remembering these targets on the final test, reducing the diagnosticity of target retrieval, and consequently, reducing JOL accuracy. Therefore, any study techniques that enhance the longevity of these targets in long-term memory should likewise enhance JOL accuracy across increasingly long retention intervals. Retrieval practice enhances target retrieval, but also target longevity. Techniques like retrieval practice might therefore lead to higher JOL accuracy than less effective techniques not only by increasing the number of targets retrieved, but also the longevity of those targets. Future investigations are needed to explore this possibility, especially because longer retention intervals are often more ecologically valid in an educational context (e.g., students using their metacognitive predictions to forecast performance on an exam a day later to motivate their study decisions).

There is another way that our results could extend to cued-recall designs. This possibility concerns situations in which people are prone to recalling false information at the time of a metacognitive prediction. Sometimes, when people are prompted to recall a target when making a prediction, they produce a false response (i.e., an error of commission). These false responses are often accompanied with high confidence, which can undermine metacognitive accuracy (e.g., Eakin and Hertzog 2012; Koriat 1993; Koriat and Levy-Sadot 2001; Krinsky and Nelson 1985; Nelson and Narens 1990; Rhodes and Tauber 2011b). Some situations are more likely to engender these issues than others. For example, Rhodes and Tauber (2011b) had participants study unrelated pairs like “Table—Cheer”. At the time of the JOL, participants were prompted not only with the cue, but also an incomplete target (e.g., Table—Ch__r) that was intended to elicit an incorrect word that was highly related to the cue (e.g., Chair). The authors also had participants study a set of control items. For the control items, the cue did not have a closely related semantic associate that would likely be produced at the time of the JOL. The authors observed higher accuracy for delayed than immediate JOLs for the control items, but not the deceptive items. Our results suggest that more efficacious encoding procedures would mitigate the issue posed by deceptive items. This is because the more effective an initial encoding technique, the more likely participants would produce the correct response, thereby reducing the probability that they would produce the deceptive lure. 

### 5.3. Noncriterial Recollection and JOL Accuracy

Our finding that noncriterial cues were not diagnostic of final-memory performance is inconsistent with the previous literature. This might owe to how we measured noncriterial cues. A cue is only diagnostic if it supports the selection of the target amongst the foils, and there are many noncriterial cues that would not aid such performance. For example, during the practice phase in which participants explained their remember/know responses, some participants correctly reported that an unretrieved target was semantically related to the cue. However, this cue may not support the selection of that target because, for weakly related items, all foils were related to the cue. It is possible that some noncriterial cues did support performance on the four-alternative forced-choice test in this experiment. However, the measurement of noncriterial cues with a remember/know task precludes an empirical evaluation of this possibility because it groups both diagnostic and non-diagnostic cues into a single measurement.

Generally, studies that found that noncriterial cues are diagnostic measured specific cues that were (a) associated with the target, and (b) useful in supporting the selection of the target amongst foils (Hertzog et al. 2010, 2014; Thomas et al. 2011, 2012; but see Isingrini et al. 2016). For example, Thomas et al. (2011) measured participants’ memory for the emotional valence (positive or negative) of unretrieved targets when making feeling-of-knowing judgments. The options on the final six-alternative forced-choice test featured both positively- and negatively-valenced words, and thus remembering valence would help performance by eliminating foils. Similarly, Hertzog et al. (2014) measured participants’ memory of the mediator images that they generated during the encoding of word pairs when making feeling-of-knowing judgments. Word pairs in that experiment either consisted of two abstract or two concrete nouns, and mediators for these pairs generally reflected the nature of the word pairs. Thus, remembering the mediator image likely cued participants into what type of target word they would need to select. As with Thomas et al. (2011), options on the final four-alternative forced-choice test featured both abstract and concrete nouns, thus rendering recollection of the mediator useful in selecting the target by eliminating foils. 

### 5.4. Limitations

One limitation of the present work is that a given target did not appear in both an unrelated and a related pair. In order words, the targets were different across item types. For example, the target “Castle” in the related pair “Throne—Castle” did not appear in any unrelated pair. Consequently, some of the differences in performance with unrelated and related items could be attributed to differences in the semantic properties of the targets rather than the intended manipulation. Even though we attempted to minimize differences in semantic properties of targets across the conditions, these differences could not be completely abolished because the targets were not identical (for a discussion, see Rhodes 2016). One way of dealing with this issue is to counterbalance the targets to ensure they appear equally often in each condition (see, Castel et al. 2007; Mueller et al. 2016; Myers et al. 2020). In the present study, this would have conflicted with our effort to make sure that the cues of one pair were not related to the targets and foils of any other pair. This is because to switch a target from one cue to another, there would necessarily need to be relationships between pairs. To illustrate, consider the pairs “Throne—Castle” and “Allergy—Divorce”. Switching the targets results in two unrelated pairs, not one pair of each type. To circumvent this issue, there would need to be pairs in each condition that would be related to one another in some way. This could be done by adding an unrelated pair like “Marriage—Duck”, so that target switching ends up with two different pair types (e.g., “Marriage—Divorce” and “Allergy—Duck”). This would also result in there being a counterbalance in which the cue of one pair would be related to the target of another pair (i.e., “Marriage—Duck” and “Allergy—Divorce”). However, for a researcher who is interested in isolating the effects of item relatedness more purely, we would recommend the counterbalancing procedure counterbalancing the targets across conditions. We would like to emphasize that we do not consider this a major limitation in the context of this study. The item type manipulation resulted in differences in memory performance across conditions, and consequently, differences in JOL accuracy, which was the intent. Moreover, many of the central findings concern analyses of effects within one condition rather than between conditions. 

### 5.5. Conclusions

These results demonstrate that the influence of study techniques on delayed-JOL accuracy is mediated by how these techniques affect the accessibility of cues. For weakly related word pairs, retrieval practice led to higher accessibility of targets than study practice, which in turn led to higher JOL accuracy. For unrelated items, there were no differences in the accessibility of targets across study-technique groups, and consequently no differences in JOL accuracy across groups. Notably, participants did utilize noncriterial cues to make their JOLs, meaning that accessibility of these cues can influence JOL accuracy if these cues are sufficiently diagnostic of final memory performance. This research suggests that accurate self-assessment of learning will depend on whether learners can access the to-be-learned information during the learning episode, and also on the careful consideration the demands of future tests and assessments. The more students understand about the relationships between to-be-remembered concepts and how they will be tested in the future, the better equipped students will be to implement the correct strategies to perform well on subsequent tests. Although learning is not only about performance on assessment metrics, understanding how the way we study interacts with what we study and how we are tested in integral to the educational experience, and self-regulated learning.

## Figures and Tables

**Table 1:** Mean remember, know, and no memory response.

Item Type	Variable	Study-Technique Group		
		SP	EE	RP
Unrelated	Remember	.40 (.27)	.45 (.23)	.40 (.21)
	Know	.35 (.19)	.31 (.17)	.44 (.18)
	No Memory	.25 (.22)	.23 (.20)	.15 (.14)
Related	Remember	.47 (.27)	.66 (.17)	.65 (.19)
	Know	.32 (.24)	.21 (.17)	.29 (.12)
	No Memory	.21 (.20)	.13 (.16)	.06 (.08)

Note. Standard deviations are given in parentheses. SP = Study Practice. EE = Elaborative Encoding. RP = Retrieval Practice.

**Table 2:** Mean cue accessibility, JOL, and final-test performance.

Item Type	Variable	Study-Technique Group		
		SP	EE	RP
Unrelated	Targets	.17 (.24)	.22 (.17)	.22 (.19)
	Noncriterial Cues	.27 (.24)	.33 (.21)	.24 (.18)
	JOL	46.58 (22.80)	53.57 (19.10)	49.31 (19.24)
	Final Test	.69 (.23)	.86 (.17)	.83 (.16)
Related	Targets	.28 (.26)	.44 (.19)	.55 (.24)
	Noncriterial Cues	.29 (.22)	.49 (.29)	.30 (.24)
	JOL	50.45 (23.47)	65.72 (16.10)	66.10 (17.51)
	Final Test	.70 (.19)	.83 (.13)	.86 (.15)

Note. Standard deviations are given in parentheses. SP = study practice. EE = elaborative encoding. RP = retrieval practice.

**Table 3:** JOL accuracy across levels of correct-guessing adjustment.

Item Type	Variable	Study-Technique Group		
		SP	EE	RP
Unrelated	JOL Acc.	.31 (.35)	.35 (.41)	.28 (.41)
	JOL Acc. (NG)	.32 (.42)	.38 (.38)	.42 (.34)
	JOL Acc. (NG, L)	.37 (.41)	.43 (.35)	.47 (.31)
Related	JOL Acc.	.31 (.27)	.45 (.32)	.58 (.30)
	JOL Acc. (NG)	.35 (.25)	.39 (.35)	.57 (.26)
	JOL Acc. (NG, L)	.40 (.27)	.49 (.28)	.60 (.26)

Note. JOL acc = JOL accuracy. JOL acc (NG) = JOL accuracy with correct guesses recoded as incorrect. JOL acc (NG, L) = JOL accuracy with correct guesses and correct low confidence responses recoded as incorrect. Standard deviations are given in parentheses.

**Table 4:** Mean utilization and diagnosticity of target retrieval and noncriterial cues.

Item Type	Variable	Study-Technique Group		
		SP	EE	RP
Unrelated	Target Utilization	.99 (.02)	.95 (.09)	.99 (.04)
	Noncrit. Utilization	.91 (.20)	.82 (.36)	.86 (.20)
	Target Diagnosticity	.22 (.34)	.07 (.40)	18 (.36)
	Noncrit. Diagnosticity	.06 (.42)	−.07 (.50)	−.11 (.50)
Related	Target Utilization	.81 (.23)	.77 (.19)	.92 (.13)
	Noncrit. Utilization	.86 (.19)	.81 (.41)	.83 (.20)
	Target Diagnosticity	.58 (.26)	.49 (.36)	.58 (.29)
	Noncrit. Diagnosticity	.02 (.49)	.08 (.48)	−.17 (.52)

Note. Values represent gamma correlations between the cue and final-test performance on an item-by-item basis. Standard deviations are given in parentheses.

**Table 5:** Experiment 1—Mediation Model Coefficients.

Predictor	Outcome Variable								
	Mediator 1: Target Accessibility			Mediator 2: Noncriterial Cue Accessibility			JOL Accuracy (Related)		
	Coeff	SE	*p*	Coeff	SE	*p*	Coeff	SE	*p*
Intercept	−.23	.04	<.001	.01	.04	.899	.45	.07	<.001
Retrieval Practice	.29	.05	<.001	.03	.06	.684	.10	.09	.295
Target Accessibility	-	-	-	-	-	-	.58	.19	.004
Noncriterial Cue Accessibility	-	-	-	-	-	-	−.02	.17	.917
	*R*^2^ = .36 *F*(1, 52) = 29.16, *p <* .001			*R*^2^ < .01 *F*(1, 52) = 0.17, *p* = .684			*R*^2^ = .32 *F*(3, 50) = 7.65, *p* = .0003		

Note. Each model represents a model in the path analysis. The final model compared retrieval practice, compared to study practice, on JOL accuracy for related items. Coeff = coefficient. SE = standard error of the mean.

**Table 6:** Mean cue accessibility, JOL, and final-test performance.

Item Type	Variable	Study-Technique Group	
		Study Practice	Retrieval Practice
Unrelated	Targets	.07 (.13)	.14 (.21)
	Noncriterial Cues	.13 (.20)	.12 (.20)
	JOL	32.46 (21.38)	35.71 (21.42)
	Final Test	.65 (.24)	.74 (.21)
Related	Targets	.32 (.26)	.56 (.26)
	Noncriterial Cues	.19 (.21)	.27 (.28)
	JOL	47.77 (23.82)	63.16 (21.38)
	Final Test	.73 (.21)	.88 (.17)

Note. Standard deviations are given in parentheses.

**Table 7:** JOL accuracy across levels of correct-guessing adjustment.

Item Type	Variable	Study-Technique Group	
		Study Practice	Retrieval Practice
Unrelated	JOL Acc.	.26 (.52)	.09 (.51)
	JOL Acc. (NG)	.25 (.53)	.06 (.44)
	JOL Acc. (NG, L)	.22 (.53)	.12 (.53)
Related	JOL Acc.	.44 (.33)	.62 (.37)
	JOL Acc. (NG)	.49 (.35)	.64 (.36)
	JOL Acc. (NG, L)	.57 (.30)	.65 (.30)

Note. JOL acc = JOL accuracy. JOL acc (NG) = JOL accuracy with correct guesses recoded as incorrect. JOL acc (NG, L) = JOL accuracy with correct guesses and correct low confidence responses recoded as incorrect. Standard deviations are given in parentheses.

**Table 8:** Mean utilization and diagnosticity of target retrieval and noncriterial cues.

Item Type	Variable	Study Practice	Retrieval Practice
Unrelated	Target Utilization	.95 (.15)	.97 (.10)
	Noncrit. Utilization	.79 (.31)	.71 (.48)
	Target Diagnosticity	.04 (.44)	.00 (.38)
	Noncrit. Diagnosticity	−.04 (.48)	−.33 (.42)
Related	Target Utilization	.83 (.23)	.85 (.32)
	Noncrit. Utilization	.89 (.34)	.78 (.39)
	Target Diagnosticity	.48 (.38)	.52 (.36)
	Noncrit. Diagnosticity	−.05 (.47)	−.22 (.42)

Note. Values represent gamma correlations between the cue and final-test performance on an item-by-item basis. Noncrit. = noncriterial cue. Standard deviations are given in parentheses.

**Table 9:** Experiment 2—Mediation Model Coefficients.

Predictor	Outcome Variable								
	Mediator 1: Target Accessibility			Mediator 2: Noncriterial Cue Accessibility			JOL Accuracy (Related)		
	Coeff	SE	*p*	Coeff	SE	*p*	Coeff	SE	*p*
Intercept	−.16	.04	<.001	−.03	.04	.476	.48	.06	<.001
Retrieval Practice	.18	.06	.004	.10	.06	.107	.14	.09	.146
Target Accessibility	-	-	-	-	-	-	.28	.19	.156
Noncriterial Cue Accessibility	-	-	-	-	-	-	−.04	.18	.825
	*R*^2^ = .123 *F*(1, 63) = 8.82, *p* = .004			*R*^2^ = .041 *F*(1, 63) = 2.67, *p* = .107			*R*^2^ = .096 *F*(3, 61) = 2.16, *p* = .102		

Note. Each model represents a model in the path analysis. The final model compared retrieval practice, compared to study practice, on JOL accuracy for related items. Coeff = coefficient. SE = standard error of the mean.

**Table 10:** Mean utilization and diagnosticity of target retrieval and noncriterial cues.

Variable	Retrieval Practice	Target Retrieval	Noncriterial Cues
Retrieval Practice	–		
Target Retrieval	.46 ***	–	
Noncriterial Cues	.09	.12	–
JOL Acc (REL)	.34 ***	.40 ***	.08

Note. * *p* < .05. ** *p* < .01. *** *p* <.001.

**Table 11:** Stage-2 Meta-analytic mediation model of study technique and JOL accuracy (related items).

Path	Estimate	LL 95% CI	UL 95% CI
1. RP → Target Accessibility	.46	.32	.60
2. Target Accessibility → JOL Accuracy	.30	.12	.49
3. Indirect Effect: RP → TA → JOL Acc.	.14	.06	.26
4. RP → NCR Accessibility	.09	−.09	.27
5. NCR → JOL Accuracy	.03	−.14	.19
6. Indirect Effect: RP → NCR → JOL Acc.	.002	−.02	.03
7. RP → JOLacc (Direct Effect)	.20	.01	.39

Note. RP = retrieval practice. NCR = noncriterial cue recall. TA = target accessibility. CI = confidence interval. LL = lower limited of the CI. UL = upper limited of the CI.

## Data Availability

Data are available at https://osf.io/j9k5b/.

## Note

1 The gamma statistic with a 2 × 2 contingency table is equivalent to Yule’s Q. With 2 × m contingency tables, gamma is not equivalent to Yule’s Q and is no longer consistent with signal-detection measures.

## Appendix A

**Item Type**	**Cue**	**Target**	**Foil 1**	**Foil 2**	**Foil 3**
Unrelated	ALLERGY	DIVORCE	BIRDS	BRAIN	DAMAGE
Unrelated	BACON	MAYOR	LINT	ELEVATOR	FRECKLE
Unrelated	BALLOON	ACRE	SPOUSE	LIBRARY	TAPE
Unrelated	BELLY	STOVE	OATS	CELLAR	EXAM
Unrelated	BEVERAGE	PHYSICS	ARTERY	TEAM	LIQUID
Unrelated	BOOZE	ICING	EMPLOYER	READER	MEETING
Unrelated	BREEZE	TOASTER	PUPPY	FEVER	POND
Unrelated	BUTLER	SEASHORE	PLAQUE	CUSTARD	DUNE
Unrelated	COTTON	SAILING	SANDALS	TEXT	PECAN
Unrelated	CRAYON	HALO	CHORE	PEDAL	CARROTS
Unrelated	DYNASTY	GRIP	SCENE	RAISIN	SLEEVE
Unrelated	EXIT	BROOM	MULE	PILL	ANTIDOTE
Unrelated	GHOST	PORCH	SHOE	SKELETON	BEAVER
Unrelated	LAMB	PARADE	LOBSTER	COMPASS	PAINT
Unrelated	LUNG	SHADOW	SHIELD	CABOOSE	MUMMY
Unrelated	NICOTINE	PACKAGE	TURTLE	SOCCER	SPATULA
Unrelated	PLUM	HELMET	EAGLE	ORCHID	SIGNAL
Unrelated	POSSUM	GLACIER	FLOOD	CARBON	FLAVOR
Unrelated	THIEF	SNOW	ACROBAT	PARROT	LAWN
Unrelated	TROUSERS	CHEF	TRAITOR	PADDLE	PRIZE
Weakly Related	BREAD	JELLY	ROLL	SANDWICH	WHEAT
Weakly Related	CAVERN	MOUNTAIN	CABIN	HOLE	TUNNEL
Weakly Related	CHAPEL	TEMPLE	STEEPLE	PRIEST	CROSS
Weakly Related	COCOON	WORM	MOTH	NEST	SHELL
Weakly Related	COFFIN	TOMB	BURIAL	GRAVE	VAMPIRE
Weakly Related	DAGGER	BLADE	SWORD	BLOOD	MURDER
Weakly Related	DENTIST	CAVITY	OFFICE	DOCTOR	DRILL
Weakly Related	DIAMOND	EMERALD	GOLD	PEARL	RUBY
Weakly Related	DOCK	SHIP	PIER	LAKE	PORT
Weakly Related	DRESSER	CLOSET	CLOTHES	DESK	TABLE
Weakly Related	GLOBE	CIRCLE	SPHERE	EARTH	ATLAS
Weakly Related	HARP	SONG	VIOLIN	PIANO	FLUTE
Weakly Related	INFERNO	FLAME	HEAT	VOLCANO	BLAZE
Weakly Related	MARSH	WEED	JUNGLE	LAND	GRASS
Weakly Related	MUSTACHE	RAZOR	FACE	MOUTH	HAIR
Weakly Related	OREGANO	HERB	PIZZA	GARLIC	SAUCE
Weakly Related	REPTILE	FROG	MAMMAL	SCALES	LIZARD
Weakly Related	SUNRISE	MOON	MORNING	DAWN	BEACH
Weakly Related	THRONE	CASTLE	SEAT	QUEEN	CROWN
Weakly Related	TOOL	MACHINE	WRENCH	KITCHEN	SHOVEL
